# Supplementary material for: A Senescence-Like Cellular Response Inhibits Bovine Ephemeral Fever Virus Proliferation
Source: Vaccines (Basel). 2021 Jun 4;9(6):601. doi: 10.3390/vaccines9060601 (PMC8227762; doi:10.3390/vaccines9060601)
Supplement: Supplementary file 1 [file vaccines-09-00601-s001.zip › Supplementary figure1.pdf]

Supplementary figure1. Cell morphology and SA-β-gal stained images of 36 BHK-21 subclones.

Isolation labels	Cell Morphology	SA-β-gal Staining
**A1**	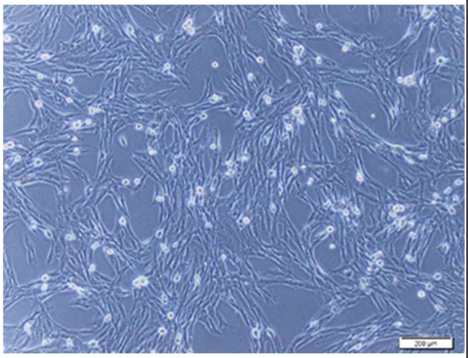	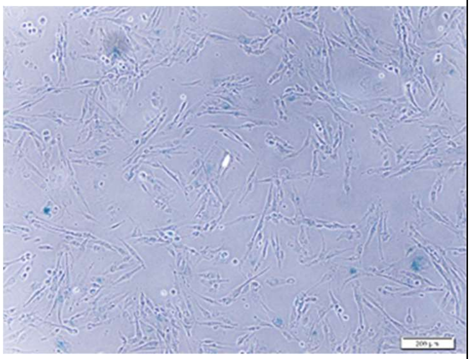
**A2**	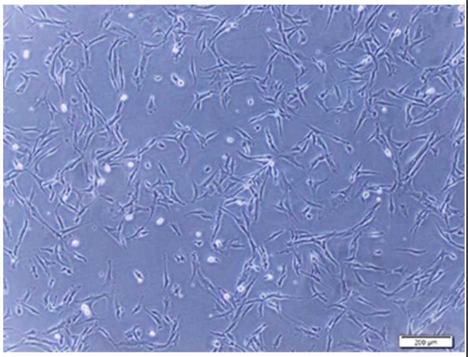	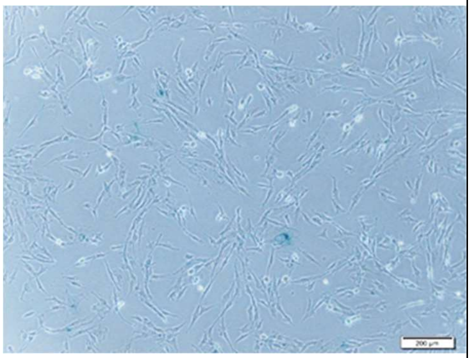
**A4**	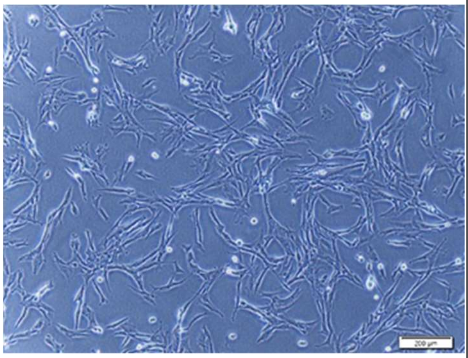	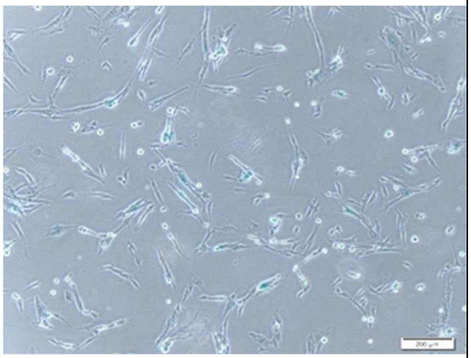
**A5**	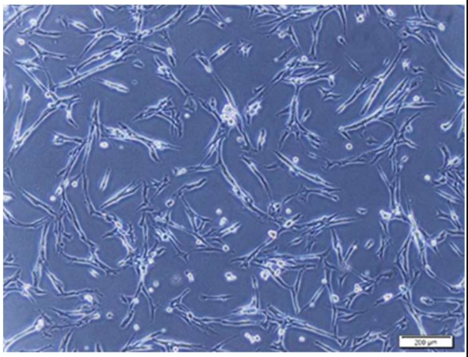	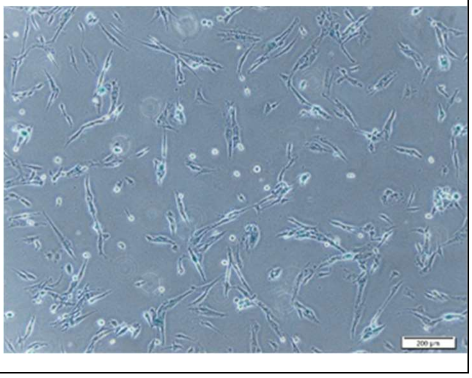

**A6**	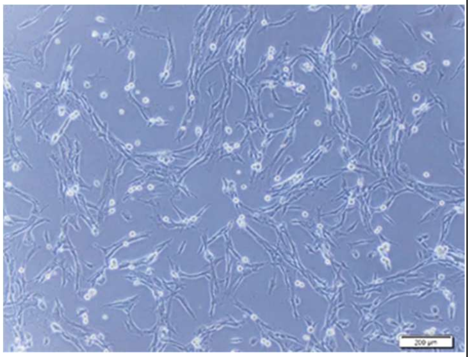	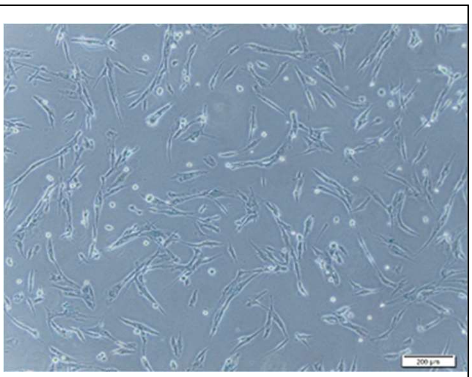
**B1**	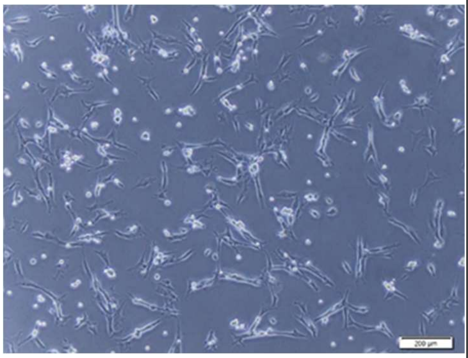	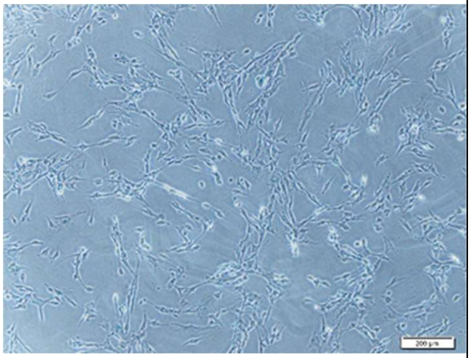
**B2**	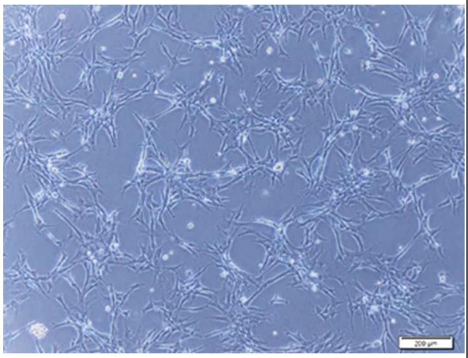	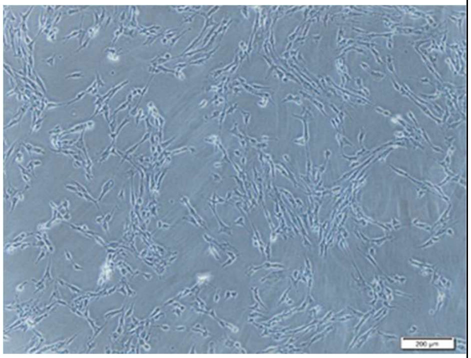
**B3**	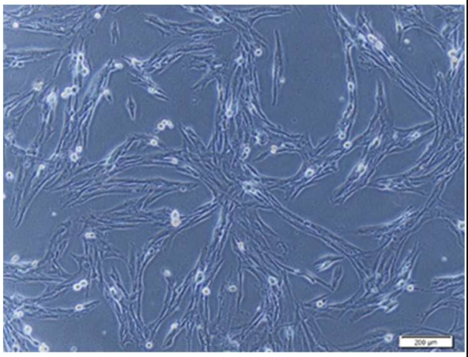	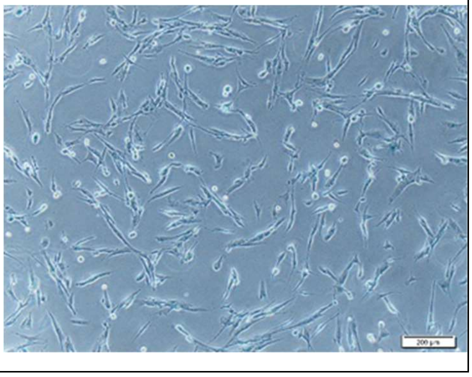

**B5**	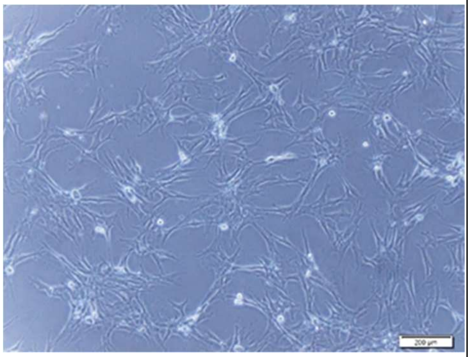	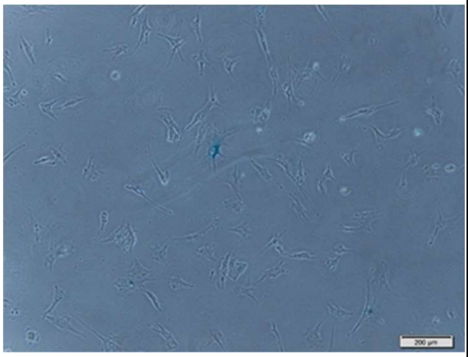
**B6**	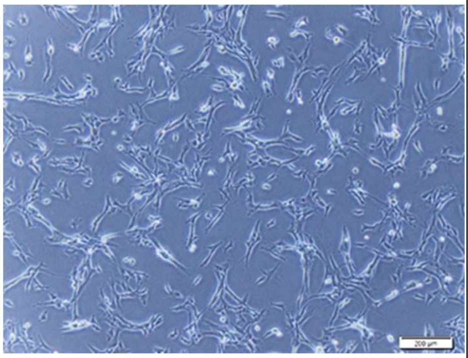	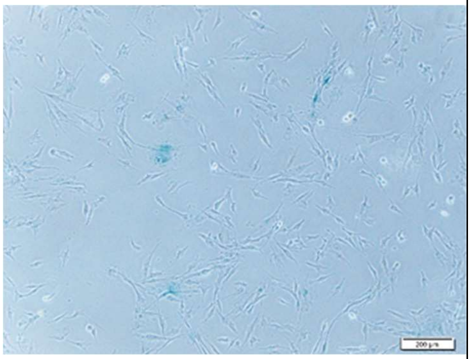
**C2**	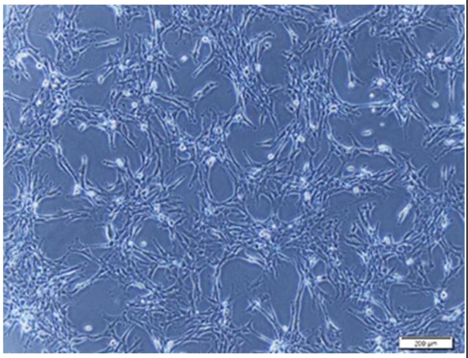	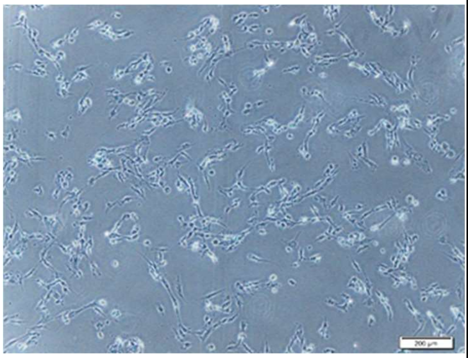
**C4**	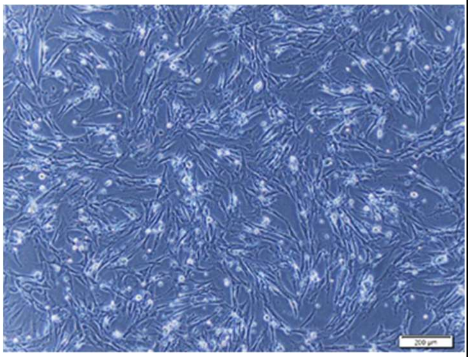	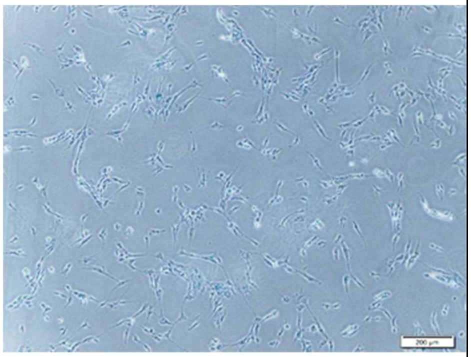

**C5**	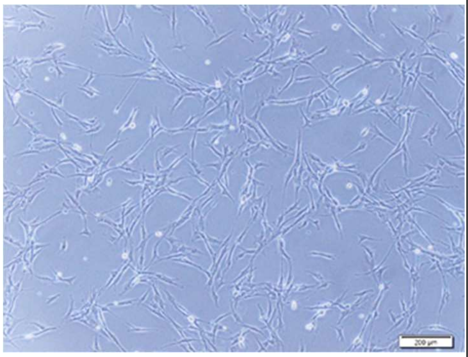	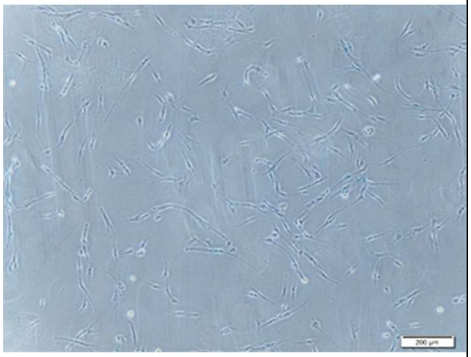
**C6**	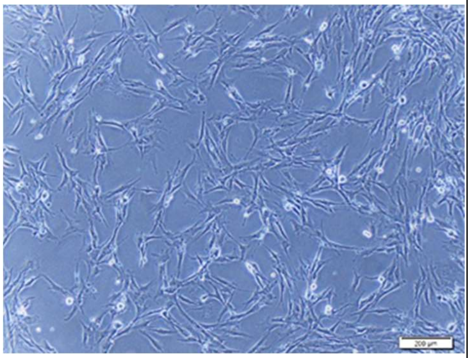	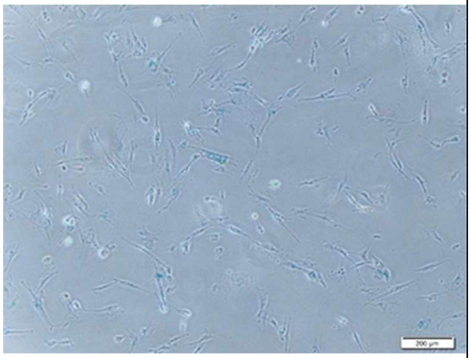
**D1**	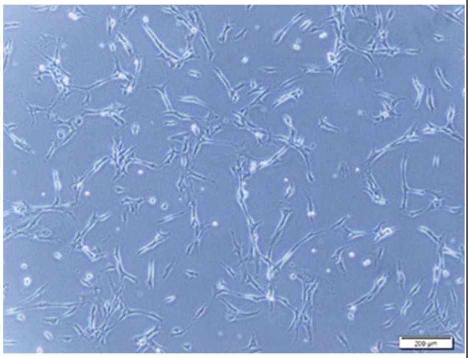	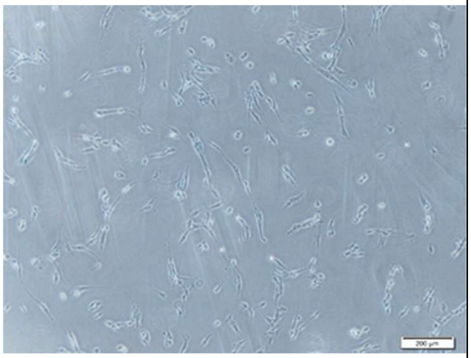
**D2**	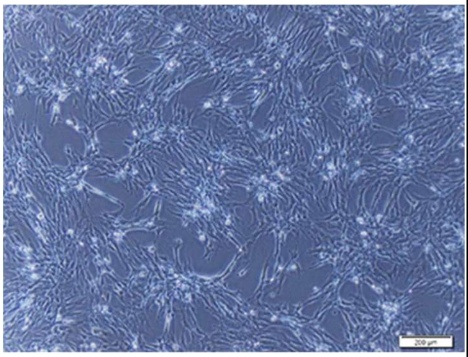	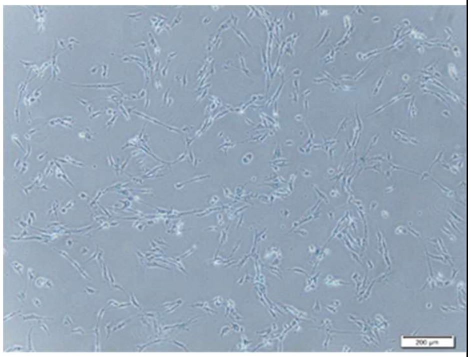

**D3**	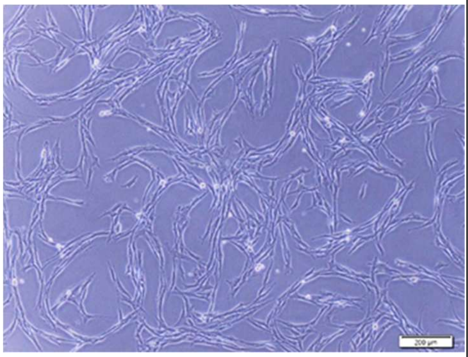	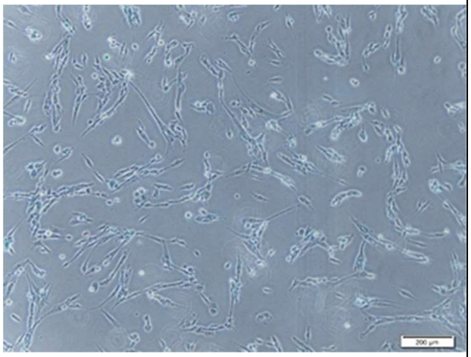
**D4**	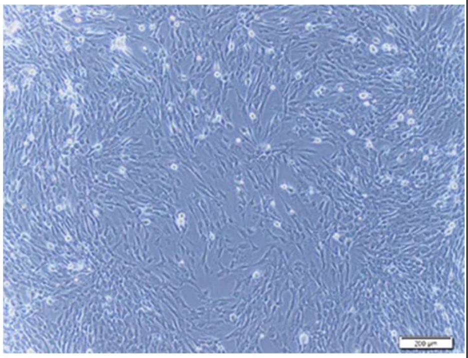	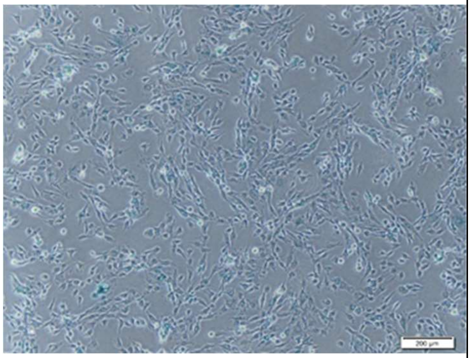
**D5**	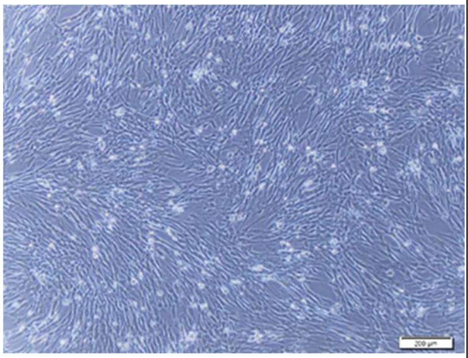	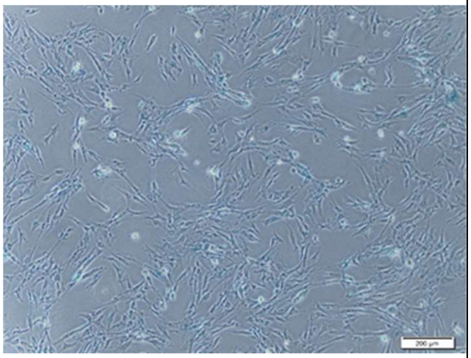
**D6**	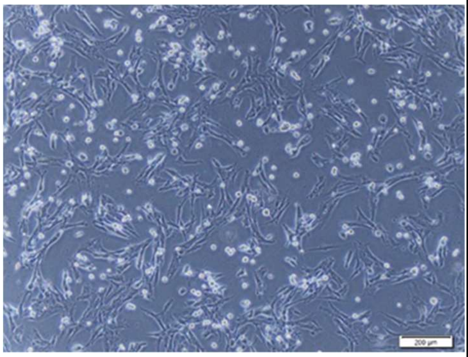	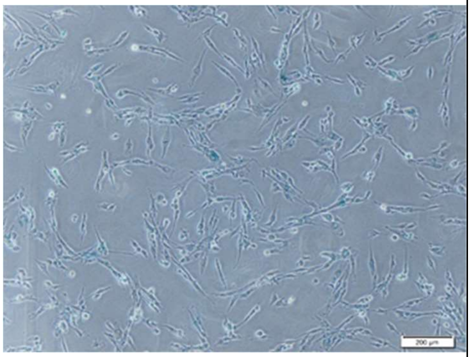

**A1-2**	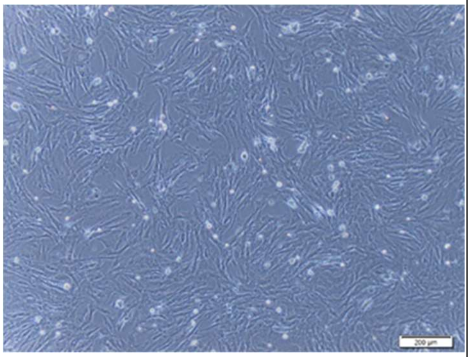	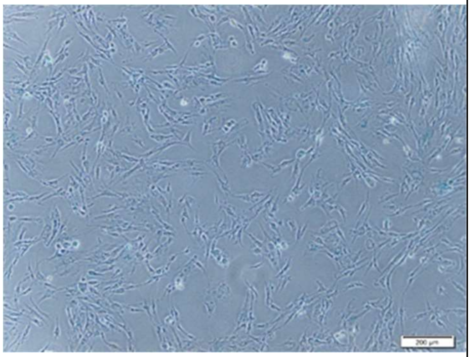
**A2-2**	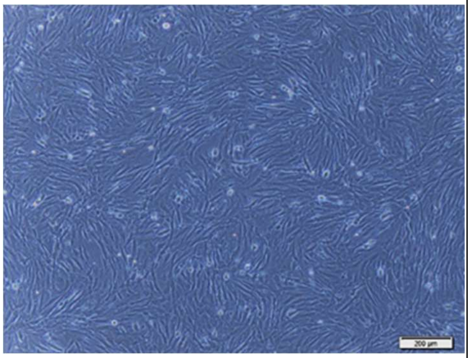	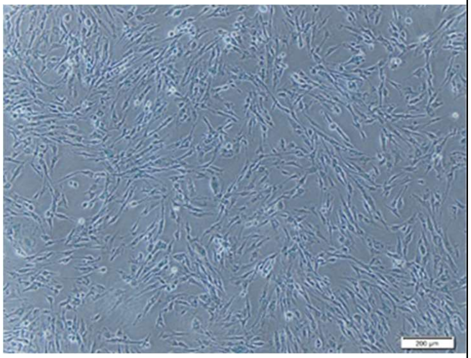
**A3-2**	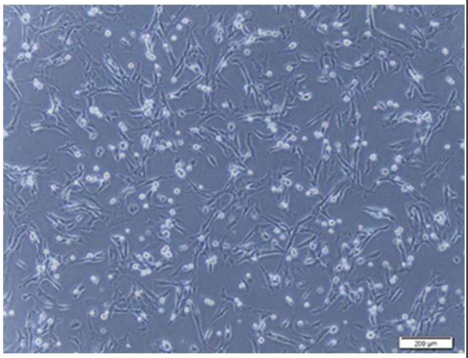	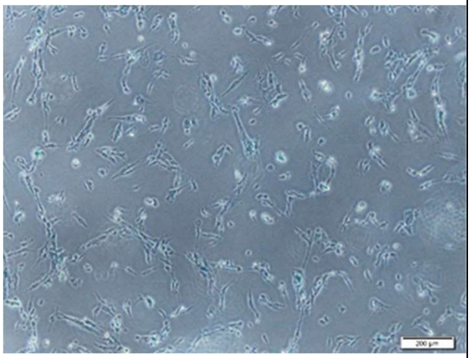
**A4-2**	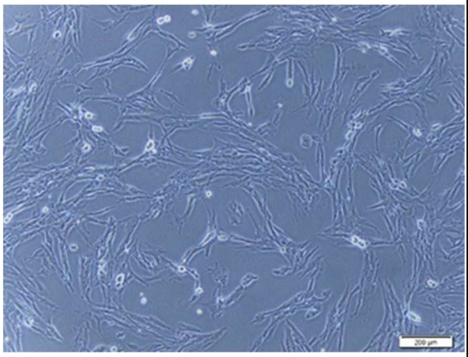	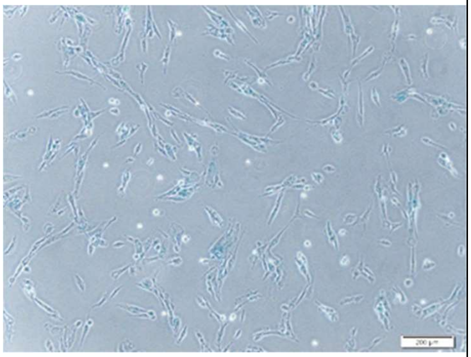

**A5-2**	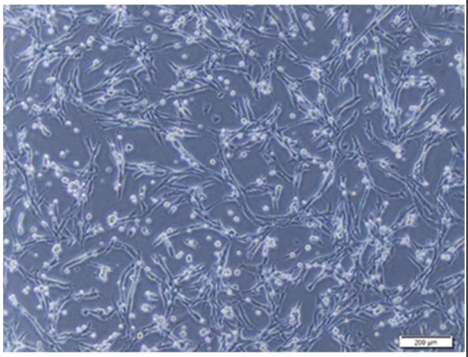	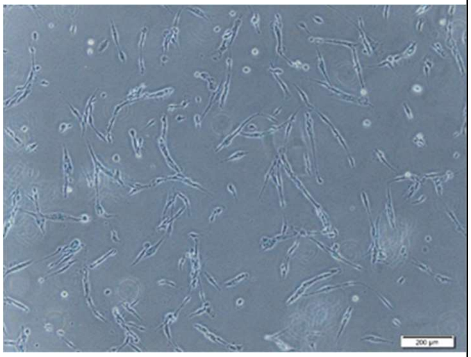
**A6-2**	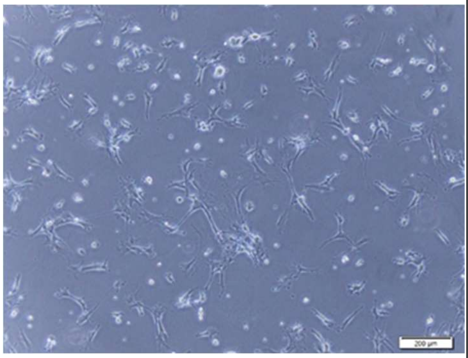	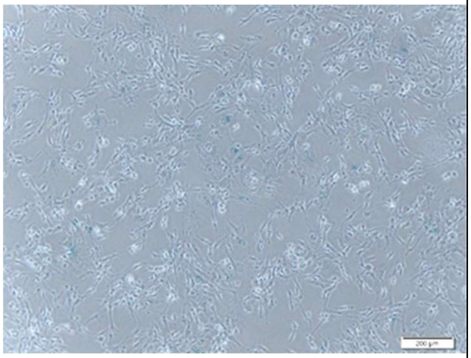
**B1-2**	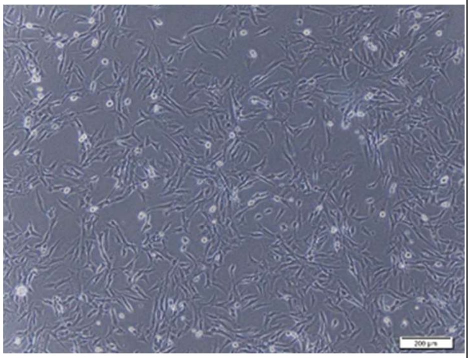	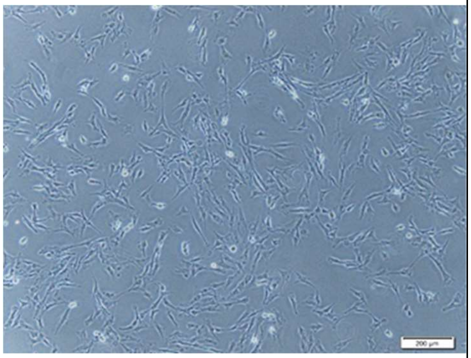
**B2-2**	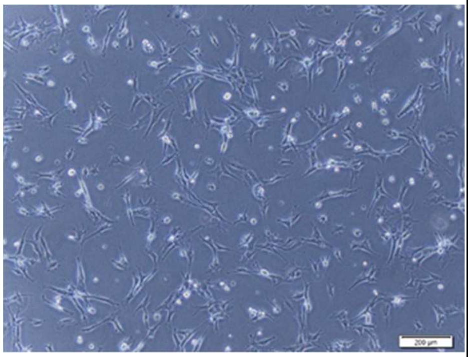	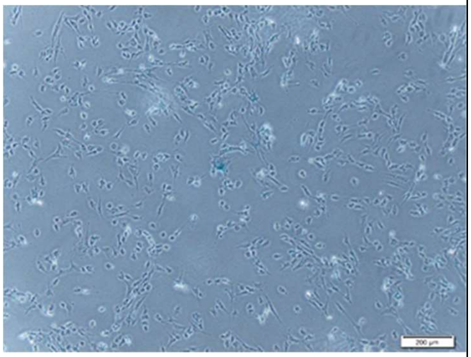

**B3-2**	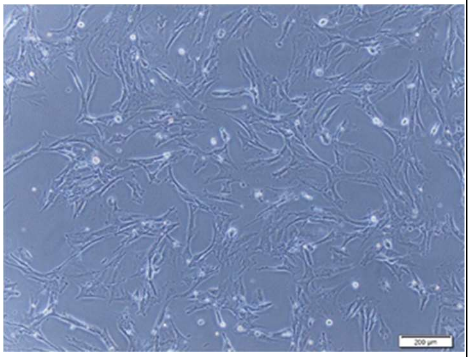	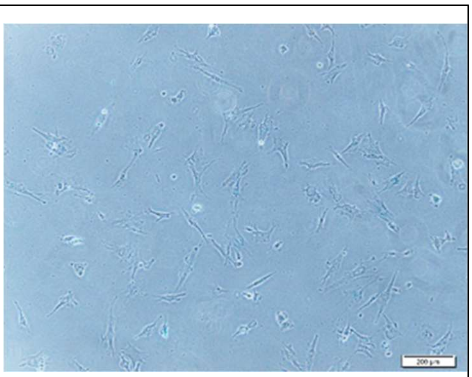
**B4-2**	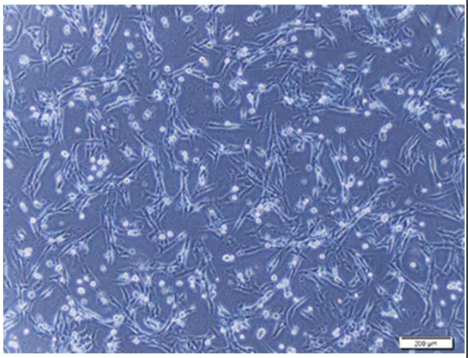	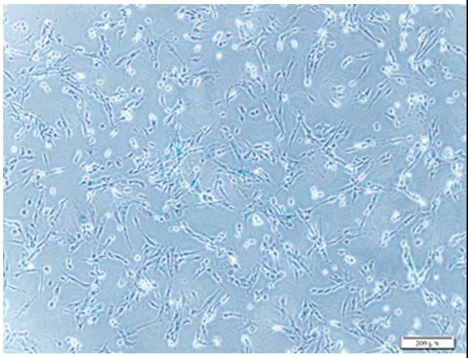
**B6-2**	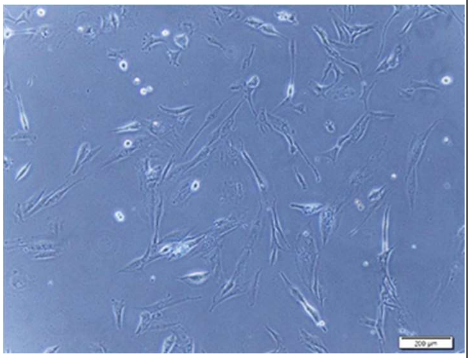	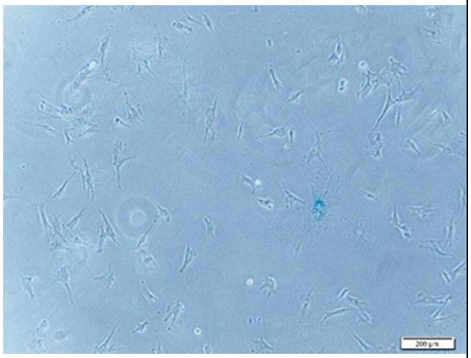
**D1-2**	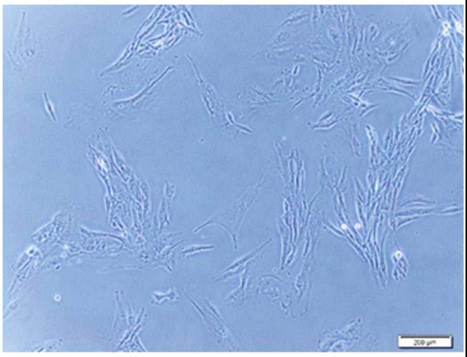	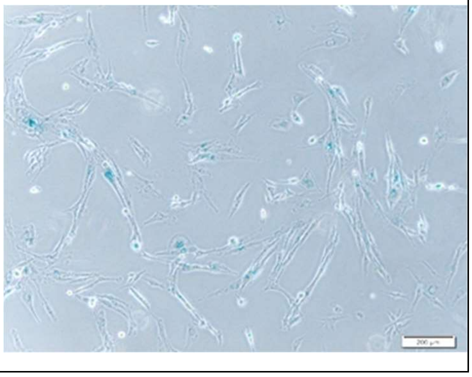

**D2-2**	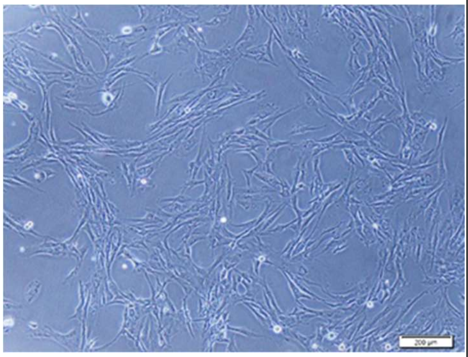	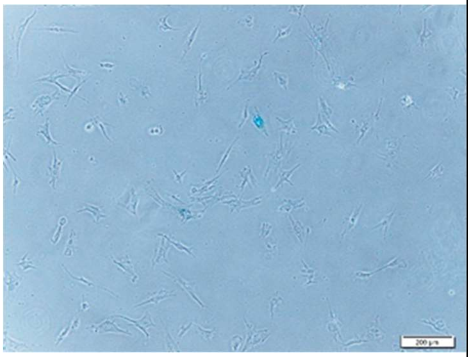
**D3-2**	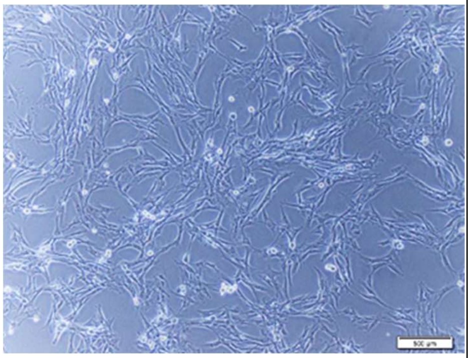	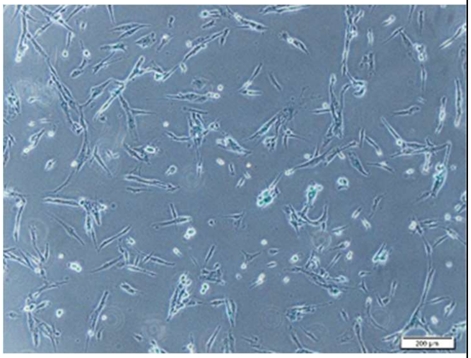
**D4-2**	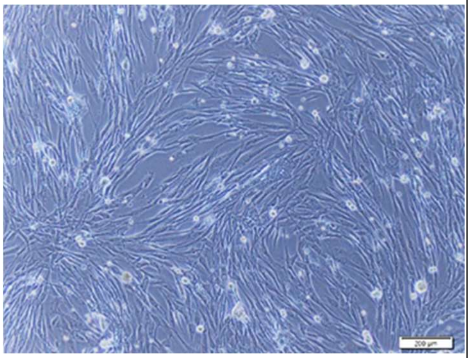	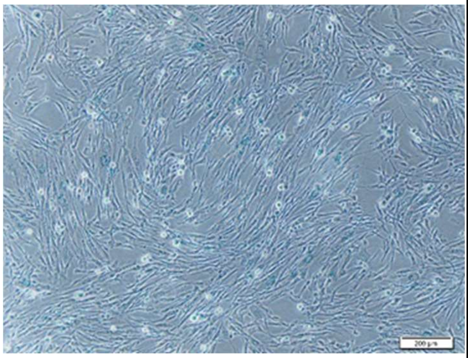
**D5-2**	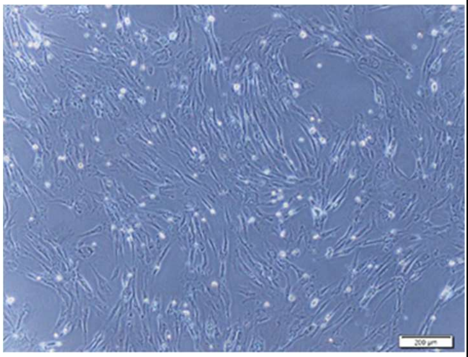	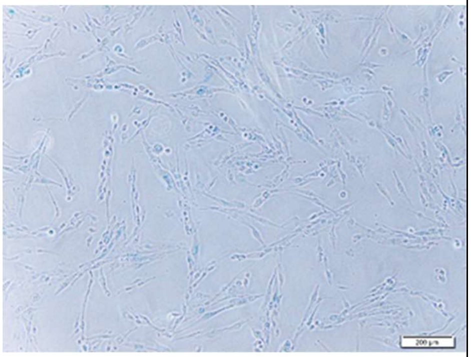
