# Supplementary material for: A Senescence-Like Cellular Response Inhibits Bovine Ephemeral Fever Virus Proliferation
Source: Vaccines (Basel). 2021 Jun 4;9(6):601. doi: 10.3390/vaccines9060601 (PMC8227762; doi:10.3390/vaccines9060601)
Supplement: Supplementary file 1 [file vaccines-09-00601-s001.zip › Supplementary figure2.pdf]

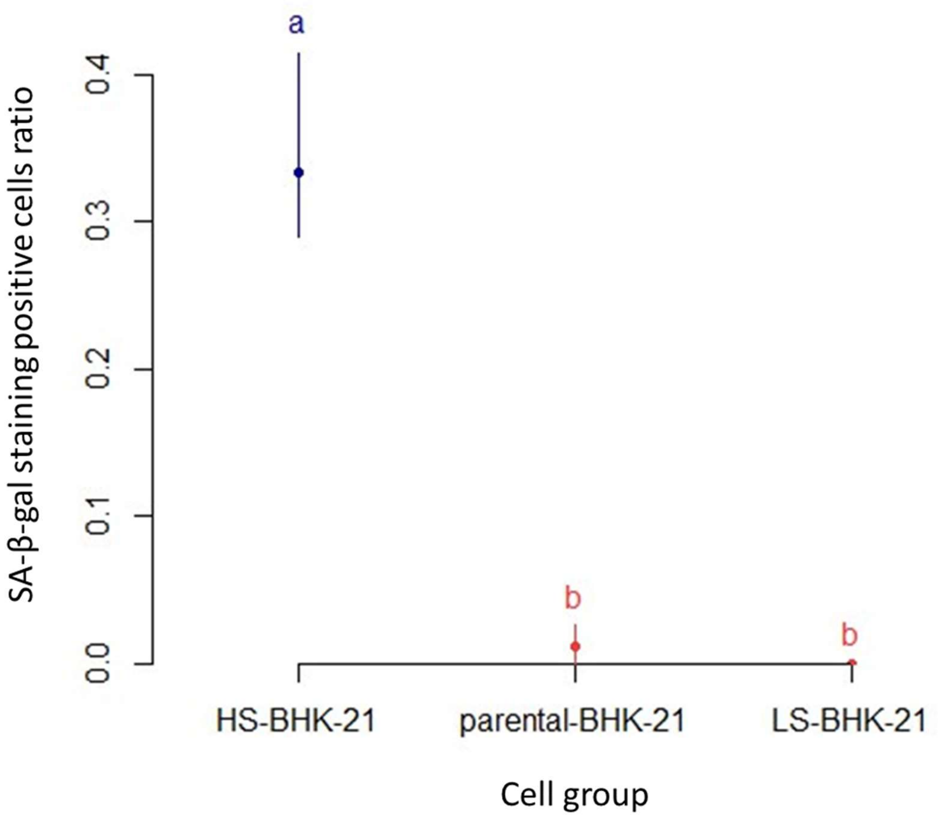

Supplementary figure 2. The SA-β-gal staining positive cell ratio in parental and subclonal BHK-21 cells. The cells were stained and image recorded. The ratio was calculated by all cells and positive cells counting in the images.
