# Supplementary material for: A Senescence-Like Cellular Response Inhibits Bovine Ephemeral Fever Virus Proliferation
Source: Vaccines (Basel). 2021 Jun 4;9(6):601. doi: 10.3390/vaccines9060601 (PMC8227762; doi:10.3390/vaccines9060601)
Supplement: Supplementary file 1 [file vaccines-09-00601-s001.zip › Supplementary figure3.pdf]

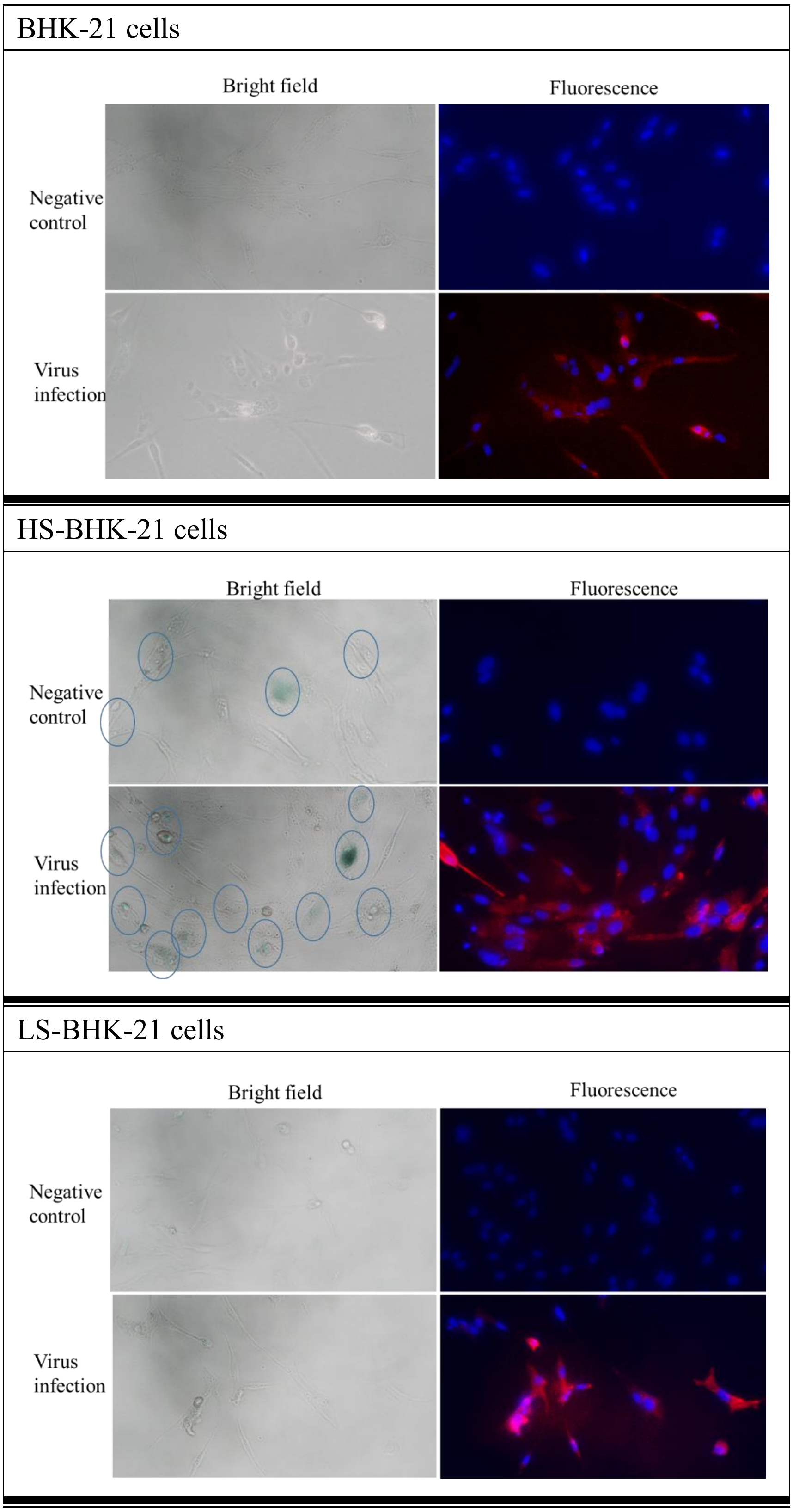

Supplementary figure 3. The BEFV can infect all three BHK-21 clonal cells. The BHK-21, HS-BHK-21, and LS-BHK-21 cells were infected by BEFV for 24hr incubation and were fixed. After SA-β-gal staining, the samples were then probed with mouse anti-BEFV N antibody and further labeled by red-fluorescence conjugated goat anti-mouse IgG antibody (#12004158, BIO-RAD, California,USA). The cells nuclear were counterstained with DAPI. The SA-β-gal staining positive cells were indicated with the blue circle.
